# Pleural Infection as the Clinical Presentation of Lung Cancer: A Case Report

**DOI:** 10.7759/cureus.91692

**Published:** 2025-09-05

**Authors:** Sarah Tseu, Marwan Abououf, Tousif Baig, Maged Hassan

**Affiliations:** 1 Respiratory Medicine, Lancashire Teaching Hospitals and NHS Foundation Trust, Preston, GBR; 2 Chest Diseases, Faculty of Medicine, Alexandria University, Alexandria, EGY

**Keywords:** empyema, lung cancer, pleural disease, pleural infections, thoracic ultrasound

## Abstract

We present a case of a 61-year-old female who was admitted with a four-week history of productive cough, worsening exertional dyspnea, and severe pleuritic chest pain, alongside several months of facial and neck swelling. Initial investigations revealed a right-sided encysted pleural effusion with biochemical features consistent with empyema, prompting intercostal drain insertion and intravenous antibiotics. A persistent cervical fullness prompted a bedside neck ultrasound, which demonstrated abnormal lymphadenopathy. A subsequent CT thorax revealed a right lung mass with mediastinal lymphadenopathy, bilateral adrenal metastases, and significant narrowing of the superior vena cava. Histological analysis of the supraclavicular lymph node confirmed metastatic small-cell lung carcinoma (SCLC). The patient was treated with corticosteroids and urgent thoracic radiotherapy for superior vena cava obstruction, followed by systemic palliative chemotherapy.

This case highlights a very unusual presentation of advanced SCLC masquerading as empyema and illustrates the importance of maintaining a broad differential diagnosis in patients with pleural infections, particularly when atypical features are present.

## Introduction

Lung cancer is a leading cause of cancer-related mortality worldwide, with many patients presenting at an advanced stage. While common symptoms include cough, hemoptysis, and weight loss, atypical presentations can often delay diagnosis.

Small-cell lung carcinoma (SCLC) represents about 15% of all lung cancers and is marked by an exceptionally high proliferative rate, strong predilection for early metastasis, and poor prognosis [1]. 

The incidence of pleural empyema as a primary finding in lung cancer patients is low (0.1%‐0.3%), and only a few references on its management and outcome are reported in the literature [2]. 

Here, we report a unique case of advanced SCLC presenting as a complicated pleural infection, underscoring the importance of maintaining a broad differential diagnosis in patients with persistent or atypical features of pleural infection. Purulent pleural fluid obtained by drainage or thoracentesis must always be examined because the association of malignant tumors and empyema should be taken into consideration [3].

## Case presentation

A 61-year-old female presented to the emergency department with a four-week history of productive cough and progressively worsening exertional dyspnea, associated with severe pleuritic chest pain. She also reported persistent facial and neck swelling over several months. She was previously fit and well with no significant medical history. She worked as a domestic cleaner and was able to perform her duties until two weeks before presentation. The patient was a smoker with a 30-pack-year history. Clinical examination revealed features of right pleural effusion. In addition, an enlarged lymph node was palpable in the right supraclavicular fossa. She had a temperature of 38.8 °C, blood pressure of 153/88 mmHg, and required 1 L of oxygen to maintain oxygen saturation above 94%.

**Figure 1:**
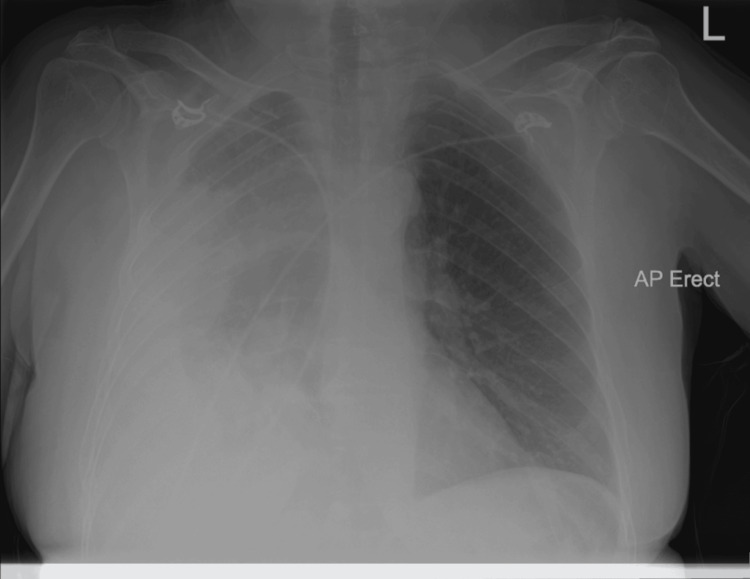
Chest X-ray performed at initial presentation showing a large right-sided pleural effusion.

The patient’s chest radiograph demonstrated a large, encysted right-sided pleural effusion with underlying consolidation (Figure 1).

Bloods test revealed markedly elevated inflammatory markers: C-reactive protein 305.2 mg/L (reference range: 0.0-5.0 mg/L), white blood count 18.03 x 10^9^/L (reference range: 4.00-11.00 x 10^9^/L), neutrophils 15.28 x 10^9^/L (reference range: 1.60-7.50 x 10^9^/L). 

**Figure 2:**
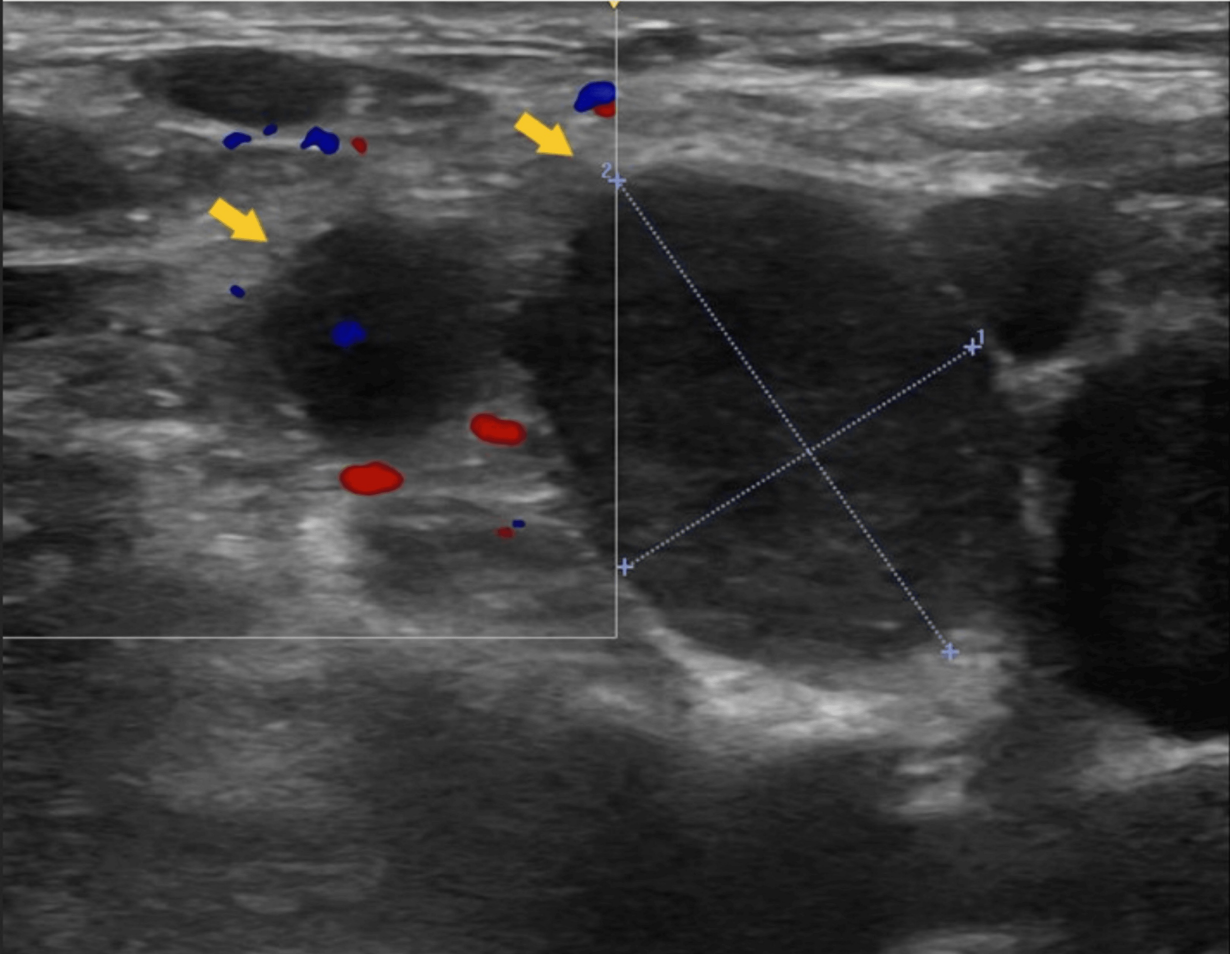
Bedside neck ultrasound revealed multiple enlarged, heterogeneous, rounded to irregularly shaped lymph nodes (yellow arrows) lacking visible hila, suggestive of a malignant etiology.

Bedside chest ultrasound demonstrated an echogenic, mildly septated right-sided pleural effusion. Diagnostic aspiration yielded yellowish, turbid fluid with a pH of 6.825, fluid glucose <1 mmol/L, and fluid lactate dehydrogenase (LDH) of 644 U/L. A diagnosis of empyema was made; an intercostal drain (ICD) was inserted, and the patient was started on intravenous antibiotics. Fluid cytology showed numerous neutrophils with scattered macrophages and lymphocytes, consistent with empyema. The patient continued to report fullness in her neck; therefore, a bedside neck ultrasound was performed, revealing multiple enlarged lymph nodes, with the largest on the right side (Figure 2). The lymph nodes appeared heterogeneous, rounded to irregular, and lacked visible hila, suggestive of a malignant etiology. 

**Figure 3:**
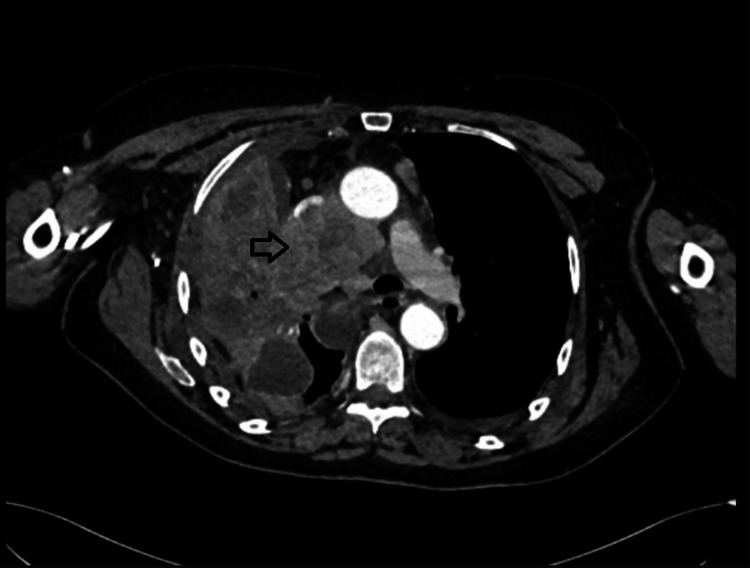
CT thorax showing a large right-sided lung mass (black arrow) with associated mediastinal adenopathy.

A computed tomography (CT) scan of the thorax, abdomen, and pelvis identified features of lung cancer with mediastinal adenopathy (Figure 3) with bilateral adrenal metastases.

**Figure 4:**
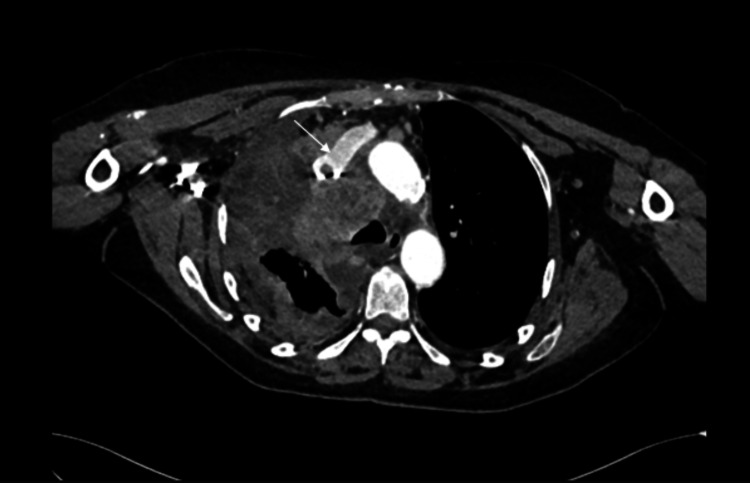
CT thorax showing a narrowed superior vena cava with tumor infiltration (white arrow).

Additionally, there was significant narrowing of the superior vena cava (SVC) with probable tumor infiltration (Figure 4). Ultrasound-guided biopsy of the right supraclavicular lymph node confirmed metastatic SCLC. 

Following consultation with the acute oncology team, the patient was started on oral dexamethasone 8 mg twice daily for SVC obstruction, which was gradually tapered after urgent thoracic radiotherapy (20 Gray [Gy] in five daily fractions over one week). Fortunately, the patient experienced significant improvement in facial and neck swelling following radiotherapy. After completing a two-week course of antibiotics and normalization of inflammatory markers, the patient began systemic anticancer treatment with palliative intent. A follow-up CT scan at three months showed complete resolution of the effusion and a substantial decrease in the size of the lung mass and lymph nodes.

## Discussion

Empyema as a presentation of lung malignancy is rare, with malignant pleural effusions being more common [4]. It is thought to result from airway obstruction by a tumor, leading to post-obstructive infection. Given the high morbidity and mortality associated with empyema, prolonged antibiotics, drainage, and in some cases surgical intervention are often required [5,6]. Clinicians should therefore maintain a broad differential, especially in patients with risk factors for lung cancer.

In this case, focused bedside neck ultrasound played a key role in raising early suspicion of malignancy through the detection of abnormal cervical lymph nodes. While ultrasound is routinely used in procedural guidance and pleural assessment, its role in detecting metastatic lymphadenopathy in patients presenting with empyema is less well reported. Prior studies have highlighted ultrasound’s value in identifying nodal metastases [7,8], and this case suggests its potential utility in expediting the diagnostic workup of lung cancer presenting atypically.

The timely diagnosis was particularly crucial given the aggressive nature of small cell carcinoma. Early recognition allowed prompt staging and oncological referral, which may improve outcomes. Although limited to a single case, this report highlights an uncommon presentation of lung cancer and suggests that focused ultrasound could serve as a useful adjunct in the early evaluation of pleural infections, warranting further study.

## Conclusions

This case highlights an exceptionally rare presentation of SCLC manifesting as empyema. The diagnostic complexity underscores the importance of considering underlying malignancy when confronted with atypical clinical features in presumed infectious processes. Focused bedside ultrasound was instrumental in revealing advanced disease, facilitating prompt oncological intervention. Ultimately, this report reinforces the need for heightened clinical vigilance and the integration of adjunctive imaging modalities to avoid missed or delayed cancer diagnoses in unusual presentations.
